# Hedonic use, stress, and life satisfaction as predictors of smartphone addiction

**DOI:** 10.1016/j.abrep.2022.100411

**Published:** 2022-01-29

**Authors:** Aleksandar Vujić, Attila Szabo

**Affiliations:** a Doctoral School of Psychology, Faculty of Education and Psychology, ELTE Eötvös Loránd University, Budapest, Hungary; b Institute of Psychology, ELTE Eötvös Loránd University, Budapest, Hungary; c Institute of Health Promotion and Sport Sciences, ELTE Eötvös Loránd University, Budapest, Hungary

**Keywords:** Dependence, Entertainment, Internet, Leisure, Mobile phone

## Abstract

This study examined the relationship between hedonic smartphone use (entertainment, social media, games), perceived life stress, and satisfaction with life with smartphone addiction (SA). We tested the connections using structural equation modeling (SEM) on questionnaire data obtained from 410 participants (73.2% women). Results indicated a good overall fit of the model (*χ^2^*(36) = 58.06, *p* = .011; CFI = 0.970, TLI = 0.954, RMSEA[90% CI] = 0.039 [0.019, 0.056], SRMR = 0.037). Perceived stress and hedonic use were positive predictors of SA (*β* = 0.264, *p* = .001 and β = 0.176, *p* = .002, respectively). Satisfaction with life did not directly predict SA, but an indirect effect, via perceived stress, was statistically significant (*β* = −0.146, *p* = .001). Women showed greater SA than men, but the effect of age was not significant. Perceived stress was negatively predicted by satisfaction with life, and positively by hedonic use. Based on the compensatory internet use theory, hedonic or non-utilitarian smartphone use might be associated with SA. The study concludes that being female, hedonic smartphone use, and perceived life stress predict SA.

## Highlights

• Hedonic smartphone use predicts the risk of smartphone addiction.
• Perceived stress predicts the risk of smartphone addiction.
• Satisfaction with life predicts indirectly and negatively the risk of smartphone addiction.
• Female gender is a direct and positive predictor of the risk smartphone addiction.
• Hedonic smartphone use positively predicts perceived stress, and life satisfaction negatively predicts perceived stress.

## 1 Introduction

Smartphones are affordable and nowadays universally used portable technological devices with access to the Internet. The estimated number of smartphone users worldwide in 2021 is > 3.8 billion, which means that nearly half of the global population owns a smartphone. This number has doubled since 2015 (O’Dea, 2021). In many parts of the world, smartphones have become an everyday necessity (Y. K. Lee et al., 2014). People benefit from smartphone use via its wide range of applications that serve various functions, many of which can directly affect a person's well-being and life satisfaction. Indeed, smartphone use can positively impact subjective well-being through applications that allow users to obtain health-related information, attention, help, and social support (Bert et al., 2014, Kang and Jung, 2014). In addition, it could improve travel experiences by making tourists better oriented feel more confident, and connected (D. Wang et al., 2016).

However, smartphone use may have negative aspects because some individuals could become overly preoccupied with it at the expense of social relations, work, study, or other important life obligations. As a result, these individuals might exhibit *problematic smartphone use* (PSU), a primary contemporary health concern. According to the *pathway model*, different patterns of smartphone use can lead to different types of PSU. Addictive use is only one of them (Billieux et al., 2015, Canale et al., 2021, Pivetta et al., 2019). This research report focuses on smartphone addiction (SA) and conceptualizes it as a component of PSU, characterized by symptoms of salience, conflict, mood alteration, withdrawal symptoms, tolerance, and relapse following the *components model of addictions* (Griffiths, 2005). Further, it also considers it a form of 'Internet addiction' because one cannot be addicted to a smartphone *per se*, but to its applications, most of which connect to the Internet (Griffiths & Szabo, 2014).

Smartphone addiction as a form of PSU could have harmful effects on physical (Inal et al., 2015, Park et al., 2015) and psychological health. For example, the SA is positively related to anxiety and depression symptoms (Chłoń-Domińczak et al., 2014, Gao et al., 2017, Hawi and Samaha, 2017, Kim et al., 2019, Rozgonjuk et al., 2018, Vahedi and Saiphoo, 2018), although this association may be inconsistent (e.g., Kuss et al., 2018). Further, PSU is also related to a decrease in sleep quality (Demirci et al., 2015), dysfunctional emotional regulation (Yildiz, 2017), lower work productivity, poorer academic performance (Duke and Montag, 2017, Hawi and Samaha, 2016, Lepp et al., 2015, Samaha and Hawi, 2016), and lower subjective well-being or quality of life (Koç and Turan, 2020, Li et al., 2020, Samaha and Hawi, 2016). A connection between PSU and increased perceived stress also exists (Elhai et al., 2017, Samaha and Hawi, 2016, Shen and Wang, 2019, Wang et al., 2015).

Some scholars suggest that excessive smartphone use should be referred to as 'problematic use' to avoid classifying it as a diagnostic entity, such as addiction (Panova and Carbonell, 2018, Tossell et al., 2015). Hence, to avoid terminological confusion, we use the term *smartphone addiction* to refer to an aspect of PSU rather than to a kind of behavioral addiction diagnosis, similar to Tossell et al. (2015). This term may be the most appropriate because excessive use, defined as frequent and voluminous, can imply addictive use (Lin et al., 2015). However, excessive use *cannot* always be considered addictive. Therefore, the *context* in which smartphones are used plays a vital role in developing SA.

The purposes of smartphone use vary. For example, Van Deursen et al. (2015) examined social and process use, renamed by Horwood and Anglim (2018) as 'entertainment use'. Social use predicted SA directly, while process use directly predicted habitual use and indirectly affected addictive use (Van Deursen et al., 2015). Another study supported these findings by showing that entertainment use is associated with PSU and anxiety (Elhai, Levine, et al., 2017). In this case, entertainment use, in addition to entertainment, relaxation, pastime, and gathering information, also refers to escaping from real-life problems or monotonous daily routines. The social use tackles the purpose of forming and maintaining social contacts and interactions via the smartphone without explicitly implying escapism or maladaptive coping elements (Van Deursen et al., 2015). In related research, coping motives (mood regulation, pastime), and perceived enjoyment predicted SA, while social and information-seeking motives were not significant predictors (Chen et al., 2017, Zhang et al., 2014). Other studies also revealed the role of entertainment and escapism in PSU and specifically SA (Panova and Lleras, 2016, Shen and Wang, 2019, Wang et al., 2015). As some results suggested a moderation effect of perceived stress on the relationship between entertainment use and smartphone addiction (e.g., Wang et al., 2015), we assumed that perceived stress might explain at least one part of the relationship between hedonic smartphone use and SA. In other words, in addition to that hedonic use might influence SA directly, there might be an indirect effect, through perceived stress. This mediation effect would be in accordance with the *compensatory Internet use theory* (Kardefelt-Winther, 2014a).

Compensatory Internet use theory states that different motivations accompanied by psychosocial difficulties lead to negative consequences of Internet use, such as online gaming and social networking. One of these motivations could be escapism – the tendency to avoid real-life problems and alleviate negative emotions using activities on the Internet or entertainment. Therefore, it is possible that the relationship between hedonic motivation and SA is partly explained by the stress an individual is facing by testing the indirect effect of the motivation (i.e., hedonic use) through perceived stress. Such incentives can lead to adverse outcomes when someone experiences particular psychosocial hardship (Kardefelt-Winther, 2014a, Kardefelt-Winther, 2014b). According to the theory, problematic Internet use is seen not as compulsive or addictive behavior but rather as a compensatory behavior that may have both positive and negative consequences. The theory serves as a framework for researching SA / PSU.

We use the terms *utilitarian* and *hedonic* (use) to describe two principal smartphone usage types. Utilitarian use refers to smartphone use that serves living activities and necessities such as banking, reading e-mails, using location services, communicating, etc. On the other hand, smartphone use also has a hedonic value when the need for instant gratification drives its use. Such gratification stems from pleasure or joy derived from watching videos, playing games, watching pornography, online shopping, unwinding, self-distracting from a stressful situation, etc. (Linnhoff & Smith, 2017).

Indeed, perceived stress is positively related to SA (Chiu, 2014, Cho et al., 2017, Samaha and Hawi, 2016, Sebastian et al., 2020, Vahedi and Saiphoo, 2018, Wang et al., 2015, Zhai et al., 2020). However, longitudinal studies suggest that the relationship between SA and stress may be inconsistent. For example, a longitudinal study found no direct effect of excessive smartphone use on stress (Karsay et al., 2019). In accord with this report, another longitudinal work could not connect heightened stress to increased nomophobia after six months (Wolfers et al., 2020). Furthermore, unlike Internet addiction, another study showed that SA could not significantly predict stress, depression, anxiety, and suicidal tendencies in a regression model, although it correlated substantially with these constructs (Wan Ismail et al., 2020). A note on this conclusion is that SA cannot be separated from Internet addiction, as discussed earlier, because smartphones are merely devices used for accessing Internet-based applications.

While practical smartphone use can positively impact subjective well-being, a recent study showed that overuse and SA negatively predicted satisfaction with life (Koç & Turan, 2020). Furthermore, excessive smartphone use appears to be associated with dissatisfaction with life (Linnhoff & Smith, 2017). However, it is not easy to establish causality in this relationship. For example, a negative relationship could exist between SA and quality of life (Li et al., 2020). In contrast, Horwood and Anglim (2019) showed that satisfaction with life was not related to PSU, but entertainment use was positively associated (*r* = 0.64) with it.

Another study reported no significant relationship between SA and satisfaction with life, although the authors revealed an indirect effect of SA on it through perceived stress and academic performance (Samaha & Hawi, 2016). Yang and colleagues found no significant effect of PSU on life satisfaction in one of their models (Yang et al., 2019). One could argue that smartphone use type (purpose) and aspects of subjective well-being are essential in studying the relationship between these two constructs. The relationship between well-being and PSU could be reciprocal, so that low personal well-being might cause perceived or actual PSU (Horwood & Anglim, 2019). This proposition stems from reports that dispositional traits, such as neuroticism, can increase SA (Horwood and Anglim, 2018, Horwood and Anglim, 2019). Low self-esteem was also related to SA (Koç & Turan, 2020). Based on these findings, being a component of well-being, we conjecture that low satisfaction with life could increase SA. In addition, based on the findings from Samaha & Hawi 2016, we sought to examine the mediation role of perceived stress between satisfaction with life and SA. In other words, the relationship where low life satisfaction is related to increased SA could be partially explained by the presence of a high amount of perceived stress, which would again be in line with the compensatory Internet use theory (Kardefelt-Winther, 2014a).

Some studies reported that women exhibit greater SA or PSU than men (Linnhoff and Smith, 2017, Lopez-Fernandez et al., 2017, Moreno-Guerrero et al., 2020, Van Deursen et al., 2015), but contrary evidence also exists (Mitchell & Hussain, 2018). Therefore, the possible gender differences in SA are equivocal. As for age, some studies found a negative relationship between age and PSU or the SA (Mitchell and Hussain, 2018, Roser et al., 2016). A study examining a large sample also provided relatively solid evidence for preschool children and young adults who reported the highest level of SA (Csibi et al., 2019). However, this inverse relationship cannot be consistently demonstrated (Moreno-Guerrero et al., 2020, Kuss et al., 2018). Based on the bulk of the extant literature, we conjecture that women exhibit a greater SA than men and that age is negatively related to SA.

We believe that this study will contribute to a better understanding of the relationship between hedonic smartphone use and SA. Unlike the study by Wang et al. (2015), where the *moderation* of perceived stress on the relation between entertainment/escapism motive and SA was examined, we have used the structural equation model inspecting the *mediation* effects of perceived stress. Previous studies used samples of Chinese college students (Chen et al., 2017, Shen and Wang, 2019, Wang et al., 2015). In this study, we examined a sample of adults from different segments and age groups of a mainly European population, making the results more generalizable. Furthermore, we tried to keep the operationalization of the hedonic use as simple as possible by assessing it with a single item, asking the participants to express *themselves* in terms of percentage of overall use. Another research used a regression analysis to examine the predictive power of different application categories on SA and life satisfaction (Linnhoff & Smith, 2017). However, as these authors point out, a particular application can belong to more than one category since it can be used for different motives. Therefore, we abandoned this approach and asked the participants about their appraisal of the hedonic motive for smartphone use, not particular applications or application categories. Finally, not examined in past works, we tested the proposition of Horwood and Anglim (2019) to obtain insight into how satisfaction with life might affect the SA.

**Fig. 1:**
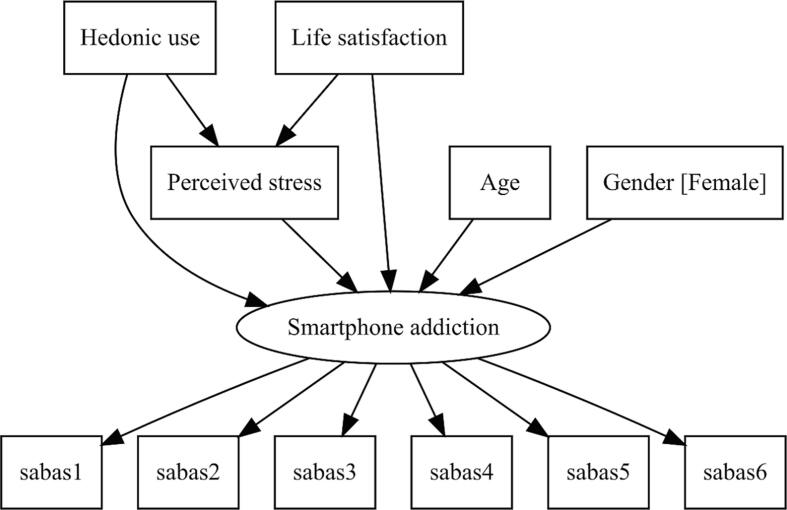
A conceptual model.

The objectives of the current study are to test the research hypotheses that perceived stress and the hedonic use of smartphones are positive predictors of SA, and that satisfaction with life will negatively predict SA. Furthermore, we expect positive indirect effect of hedonic use on SA, through perceived stress, and a negative indirect effect of satisfaction with life on SA, through perceived stress. We also propose that age is a negative predictor of SA. Finally, as mentioned above, we conjecture that women exhibit higher SA than men. The conceptual model is shown in Fig. 1.

## 2 Method

### 2.1 Participants

Participants were 410 adult volunteers, of whom 300 (73.2%) were women aged between 18 and 77 years, *M* = 32.32, *Mdn* = 30 (*SD* = ± 10.85). Initially, there were 469 respondents, but we have removed incomplete responses. Additionally, we also removed data obtained from two participants who did not use a smartphone. Twenty-four (23.9%) percent of the final sample completed high school, 56.3% had a university, and 19.8% had postgraduate degrees. They completed the study in English. Having a good mastery of English was explicitly required in the call for participants posted on various social media (see Procedure section).

### 2.2 Ethics

The Research Ethics Board of the Faculty of Education and Psychology at ELTE Eötvös Loránd University granted ethical clearance (Certificate Number 2020/306) for the current study. All participants read and consented to anonymous participation by answering with 'Yes' to the question if they were willing to participate.

### 2.3 Materials

Demographic questions asked participants their gender, age, and education level. In addition, two single-item frequency scales asked the percent of time participants access the Internet via smartphones and the percent of the time they use their devices for *utilitarian* and *hedonic* purposes. Finally, we collected responses to three questionnaires described below. It took approximately eight to ten minutes to complete all the questionnaires.

***Smartphone Application-Based Addiction Scale*.** (SABAS; Csibi et al., 2018). The SABAS is a six-item, one-dimensional instrument intended to assess addiction symptoms related to smartphone application use based on the *components model of addiction* (Griffiths, 2005). It is rated on a six-point Likert scale (ranging from 1 = *strongly disagree* to 6 = *strongly agree*). The SABAS has been validated in several languages, including Hungarian (Csibi et al., 2016), English (Csibi et al., 2018), and Chinese, 2020 (Leung et al., 2020, Yam et al., 2019). The reported reliability of the English version was good (Cronbach's alpha [*α*] = 0.81). In the current study, the internal reliability of the SABAS was 0.75.

***Perceived Stress Scale*** (PSS-4; Cohen et al., 1983). This scale assesses perceived stress in the past month. It is rated on a 5-point Likert scale (1 = *never* to 5 = *very often*). The reliability of PSS-4 in previous research ranged from Cronbach's α 0.67 to 0.82 (E. H. Lee, 2012). In the current study, the internal reliability of the PSS-4 was.74.

***Satisfaction With Life Scale*** (SWLS; Diener et al., 1985). This instrument is a five-item tool rated on a 7-point Likert scale, ranging from (1 = *strongly disagree* to 7 = *strongly agree*). The initially reported internal consistency of the scale was α = 0.87 (Diener et al., 1985). In the current study, the internal reliability of the SWLS was 0.85.

***Device type for Internet access***. Participants were required to report their best estimate of the percent (time) that they use various devices to access the Internet. The devices listed were smartphones, tablets, desktop computers, and laptop computers. The percentages needed to add up to 100%.

***Hedonic and Utilitarian Use***. Participants reported their best estimate of the percent of time they use their smartphones for *hedonic* purposes (including entertainment, surfing on social media, playing games, etc.). They also estimated the percent of time spent with utilitarian purposes (studying, work, e-banking, paying bills, participating in online work/study meetings, etc.). These percentages had to add up to 100. Since the two forms of use are mutually exclusive, we only analyzed *hedonic* use on the utilitarian-hedonic continuum. These two questions referred to the overall use in general. We note that although the question was phrased using the 'percentage of time' term, these are not actual percentages calculated from the frequency of use, but simply self-reported use. Therefore, this scale is no different than the Likert scale, and it is treated as interval, given the large amount of answer points.

### 2.4 Procedure

Respondents participated in the current study by anonymously filling out questionnaires on the Qualtrics research platform (Qualtrics, 2017), having a unique uniform resource locator (URL). Call for participants was posted on various social networking sites, such as Facebook, Twitter, LinkedIn, and applications such as WhatsApp and Instagram. Participation in the research was anonymous, with no material compensation offered to the participants, who could withdraw from the study at any time without consequences. Before proceeding with the data analyses, we checked the data validity by examining the minimum duration of completion (realistically enough time), meeting the criteria for participation (aged 18 years or over and user of a smartphone), and the answers' completeness.

### 2.5 Data analysis

Data were analyzed using IBM SPSS 27 (IBM Corp., 2020) and R version 3.6.2 (R Core Team, 2021). Structural equation modeling (SEM) was performed with the 'lavaan' and 'lavaanPlot' packages (Lishinski, 2021, Rosseel, 2012). The hypotheses were tested in a single structural model. A latent variable represented SA: all six SABAS items loaded on a single factor. Perceived stress and satisfaction with life were entered as average scores of the respective items and hedonic use as a single item, divided by 10 to lower the variance range (Kline, 2016). The guidelines for good model fit indices were: for RMSEA ≤ 0.06, for CFI and TLI ≥ 0.90, and for SRMR < 0.08 (Hooper et al., 2008).

## 3 Results

### 3.1 Descriptive measures

Cronbach's alpha and McDonald's omega coefficients were computed on 412 cases. Reliabilities for SABAS (*α* = 0.75, *ω* = 0.76) and PSS-4 (*α* = 0.74, *ω* = 0.72) are acceptable, while for SWLS (*α* = 0.85, *ω* = 0.85) is excellent. The descriptive statistics of the scales are shown in Table 1. Table 2 shows the zero-order correlation coefficients between the continuous variables used in the analysis. As for smartphone use, 7.28% of the participants reported low (0–20% of the time) frequency of smartphone use as a means to access the Internet relative to tablets, desktop computers, and laptop computers, including the two participants who reported no use of smartphone at all. Next, 16.02% reported medium–low frequency (20–40%), 28.88% reported medium frequency (40–60%), 30.83% high frequency (60–80%), and finally 16.99% reported very high frequency for using a smartphone to access the Internet (80–100%).

**Table 1:** Descriptive statistics of the various measures and items.

	*M*	*Mdn*	*SD*	skewness	kurtosis
Perceived stress	2.69	2.75	0.75	0.21	−0.08
Life satisfaction	4.59	4.80	1.22	−0.51	−0.28
Smartphone addiction (SA)	2.81	2.67	0.91	0.24	−0.47
Hedonic use	56.11	60.00	25.00	−0.10	−0.75
SABAS item 1	2.64	2.00	1.37	0.51	−0.81
SABAS item 2	2.12	2.00	1.23	1.16	0.57
SABAS item 3	3.18	3.00	1.49	−0.01	−1.29
SABAS item 4	3.30	3.00	1.39	0.04	−1.04
SABAS item 5	2.58	2.00	1.26	0.57	−0.63
SABAS item 6	3.07	3.00	1.44	0.29	−1.11

*Note*. *M* = mean. *Mdn* = Median. *SD* = standard deviation. SABAS 1 to SABAS 6 are the items of the SABAS questionnaire.

**Table 2:** Zero-order correlation coefficients between age, perceived stress, satisfaction with life, hedonic smartphone use, and smartphone addiction.

	1	2	3	4	5
1 Age	–				
2 Perceived stress	−0.07	–			
3 Life satisfaction	0.01	−0.56^***^	–		
4 Smartphone addiction (SA)	−0.10*	0.31^***^	−0.23^***^	–	
5 Hedonic use	−0.22^***^	0.15^**^	−0.12*	0.23^***^	–

*Note*. *p < .05. **p < .01. ***p < .001.

As seen in Table 2, SA shows the strongest positive correlation with perceived stress, followed by hedonic use, and a negative correlation with satisfaction with life. The highest correlation emerged between perceived stress and satisfaction with life.

### 3.2 Structural equation model

**Fig. 2:**
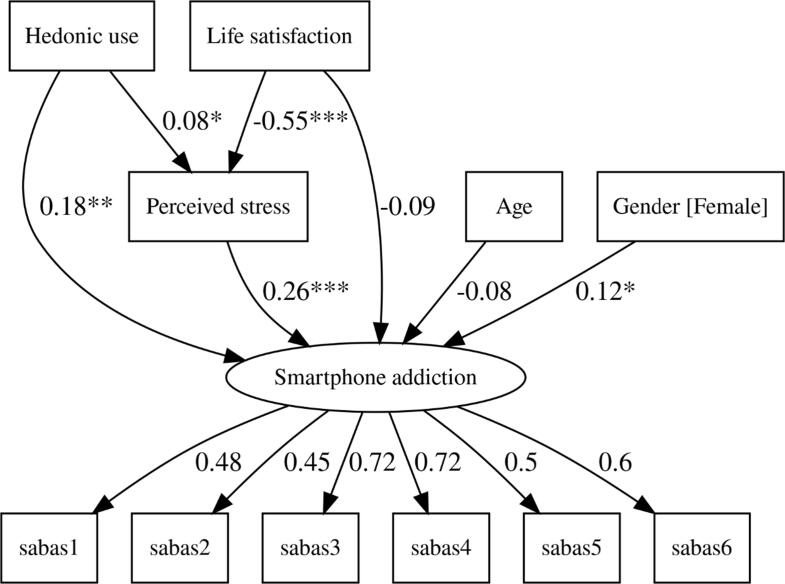
Path diagram of the hypothesized model, with standardized path coefficients Note. Uniqueness, disturbance, and covariance arrows are omitted from the diagram for the sake of clarity. All exogenous variables are allowed to covary. Standardized regression coefficients (next to the arrows going to perceived stress variable and smartphone addiction factor) and factor loadings (next to the arrows going from smartphone addiction factor to individual SABAS items) are presented. All factor loadings are significant. *p < .05, **p < .01, ***p < .001.

There was a single latent variable (smartphone addiction) in the structural model, with six indicator variables (i.e., six SABAS items) and five manifest variables: age, gender, hedonic use, life satisfaction, and perceived stress. The model was fitted using the maximum likelihood (ML) estimation, with bootstrapped standard errors, and showed a good global fit, *χ^2^*(36) = 58.06, *p* = .011; CFI = 0.970, TLI = 0.954, RMSEA[90% CI] = 0.039 [0.019, 0.056], SRMR = 0.037. Although the chi-square test statistic was significant, this is due to great sensitivity to the sample size. Bootstrapping (n = 2000) was performed to obtain 95% confidence intervals of the parameters, based on the adjusted bootstrap percentile method. The model diagram is presented in Fig. 2.

Gender, perceived stress, and hedonic use were significant predictors of SA. As the gender variable was coded 0 = males, 1 = females, it means that being a female positively predicted the SA. Perceived stress and hedonic use also predicted the outcome in a positive direction. Next, perceived stress was positively predicted by hedonic use and negatively by life satisfaction.

Although the direct effect of satisfaction with life on SA was not significant, there was a significant negative indirect effect of satisfaction with life on SA through perceived stress. An indirect effect of hedonic use on SA was not significant, but the confidence interval did not include zero, despite the lower bound being very close to zero. The regression coefficients with confidence intervals are shown in Table 3. All variables explained 18.1% of the variance of smartphone addiction, while satisfaction with life and hedonic use explained 32.4% of the variance in perceived stress.

**Table 3:** Unstandardized regression coefficients, with standard errors, z-values, significance, and confidence intervals of the coefficients.

	Parameters		*B*	*SE*	*z*	*p*	95% *CI*	
							*LL*	*UL*
Direct effects								
	Smartphone addiction (SA)							
		Hedonic use	0.046	0.015	3.023	0.003	0.017	0.078
		Life satisfaction	−0.051	0.041	−1.278	0.201	−0.127	0.026
		Stress	0.233	0.067	3.480	0.001	0.106	0.368
		Age	−0.005	0.004	−1.406	0.160	−0.012	0.002
		Gender [Female]	0.176	0.085	2.067	0.039	0.010	0.349
	Stress							
		Hedonic use	0.025	0.012	2.054	0.040	0.001	0.048
		Life satisfaction	−0.337	0.026	−13.069	<0.001	−0.386	−0.284
Indirect effects								
	Smartphone addiction (SA)							
		Hedonic use	0.006	0.003	1.651	0.099	0.001	0.013
		Life satisfaction	−0.079	0.023	−3.478	0.001	−0.125	−0.036
Covariances								
	Hedonic use							
		Life satisfaction	−0.368	0.161	−2.288	0.022	−0.688	−0.043
		Gender [Female]	0.095	0.055	1.735	0.083	−0.012	0.204
		Age	−6.062	1.465	−4.139	<0.001	−9.047	−3.419
	Life satisfaction							
		Gender [Female]	0.053	0.027	1.967	0.049	0.002	0.111
		Age	0.159	0.641	0.248	0.804	−1.067	1.439
	Gender [Female]							
		Age	0.455	0.209	2.175	0.030	0.046	0.857

*Note*. *B* = unstandardized coefficient. *SE* = standard error of the coefficient. *z* = *z*-test value. *p*: *p*-value. *CI* = bias-corrected confidence interval. *LL* = lower limit of the confidence interval. *UL* = upper limit of confidence interval.

## 4 Discussion

This cross-sectional study reveals that hedonic smartphone use predicts the SA. This finding supports two previous reports that higher entertainment-oriented smartphone use will more likely lead to PSU or SA than non-entertainment or utilitarian use (Jeong et al., 2016; S. J. Lee et al., 2016). One possible explanation could be based on the *uses and gratification theory* (Katz et al., 1973), which states that people choose which content to consume based on their personal needs. For example, hedonic needs can involve socialization, mood regulation, sexual gratification, or entertainment (Shen & Wang, 2019). A common feature of most, if not all, addictions is that when an instant reward is accessible, it is easier for an individual to become addicted to the behavior (S. J. Lee et al., 2016). According to the *compensatory Internet use theory*, problematic Internet use, being directly related to and inseparable from PSU, comes from maladaptive coping. For example, a person uses the Internet/smartphone to escape real-life problems or relieve stress (Kardefelt-Winther, 2014a). Hedonic smartphone use can be a route of escape yielding pain relief (or distraction from a problem, uncontrollable situation, distress) through gratification, via watching videos, pornography, playing various games, listening to music, using social networks, and gathering information through social media and news, related to SA. Overall, supporting the results of previous research, this study confirms hedonic smartphone use as a predictor of SA.

We also found that perceived stress is a predictor of the risk of SA. This finding also supports previous reports (Samaha and Hawi, 2016, Wang et al., 2015). It may also be related to the compensatory Internet use theory. Individuals who consider their situation unmanageable will need to use smartphones to escape or alleviate stress. In this case, a higher stress level may trigger increased smartphone use.

The results further suggest that perceived stress can explain a part of the relationship between hedonic use and SA since there was an indirect effect of hedonic use on SA through perceived stress. However, it should be noted that the effect was not statistically significant, but the confidence interval indicated that some effect might be present. Nonetheless, if the effect exists, it appears to be very small. This finding implies that other factors could also mediate the relationship between hedonic use and SA. For example, the use of smartphones for hedonic purposes may lead to increased stress. Using a smartphone for entertainment may harm productivity and daily life, such as a person not fulfilling obligations and tasks at all or in time, leading to increased perceived stress (the perceived stress is conceptualized as a feeling of the lack of control over life events). In turn, perceived stress may lead to greater SA since the smartphone is used as a tool to alleviate negative emotions. However, the interrelation of use motivation, perceived stress, life satisfaction, and SA is most likely reciprocal (Horwood and Anglim, 2019, Samaha and Hawi, 2016).

Satisfaction with life did not predict SA directly, with all other variables included. In other words, no direct effect emerged, but there was an indirect effect of satisfaction with life on SA through perceived stress. These results suggest that the perceived stress may explain the relationship between life satisfaction and SA. People dissatisfied with their lives may experience more perceived stress and engage in maladaptive smartphone use to cope with the distress and negative affect or escape real-life problems and distressing thoughts, thus increasing SA.

In our study, the age of the participants did not predict the risk of SA. This finding contrasts some previous findings (Hussain et al., 2017, Mitchell and Hussain, 2018, Roser et al., 2016), but is in line with others (e.g., Kuss et al., 2018). However, the current results reveal a small but statistically significant negative correlation between age and SA and a stronger negative correlation with hedonic use. In both cases, the shared variance, however, is too small to be considered meaningful. Furthermore, unlike the study by Csibi et al. (2019) that tested children as young as three (3) years old, our results are based on an adult sample aged 18 years and older. Perhaps a broader age range could have produced a more accurate picture of the relationship between age and SA than the heterogeneous sample of adults studied here.

In accord with previous research, our study supports the findings that the female gender is a predictor of SA. This finding has often been reported when investigating gender differences regarding SA (Linnhoff and Smith, 2017, Lopez-Fernandez et al., 2017), but some studies could not confirm this connection (Mitchell & Hussain, 2018). An explanation could be that there are different motives for smartphone use in women and men. For example, escapism was found to be higher in women than men (Linnhoff & Smith, 2017). Furthermore, women showed a greater tendency to develop habitual or addictive smartphone use through the more prominent social use and social stress (van Deursen, et al., 2015). Thus, the current results need further scrutiny to identify the factors associated with frequently reported gender differences related to SA.

This study contributed to a better understanding of mutual relations between hedonic use purpose, perceived stress, satisfaction with life, age, gender, and smartphone addiction. First, we used a short, valid, and reliable scale, SABAS, which is based on the 'components model of addiction.' In contrast, most previous studies were based on other, not necessarily theory-driven, instruments. Next, we have further confirmed the principal postulations of the compensatory Internet use theory (Kardefelt-Winther, 2014a) on a primarily Western and wide age group sample in contrast to Chinese students examined in similar studies. Most importantly, we showed that the simple, single-item operationalization of the *smartphone use purpose* could be adopted. The subjective appraisal of *what the participants believe to be utilitarian or hedonic purpose* may be more accurate than asking them the (estimated) frequency of specific application use and then posteriorly classifying these into the respective categories because some applications might satisfy both purposes.

### 4.1 Limitations

This study has limitations that call for caution in interpreting the results. First, this study relied on a convenience sample, and the data were collected online, leaving the possibility of self-selection bias. Second, the questionnaires were in English, and we did not control for the language proficiency of the respondents. Third, the cross-sectional design limits the drawing of causal conclusions. Fourth, based on the small variances explained by the predictors (a little less than one-fifth), it is likely that a considerable proportion of variance in SA is attributable to other factors. These factors may include personality traits, various morbidities, chronic use, and psychopathological characteristics (e.g., Elhai et al., 2017, Shen and Wang, 2019, Van Deursen et al., 2015). Therefore, future research should also include these critical factors in the model, which might lead to a greater amount of explained variance in SA. Fifth, an important limitation of our work is that despite asking participants for the percent of the time of accessing the Internet via smartphones, when we examined the answers related to Internet-based application use, we assumed that such access occurred exclusively via smartphones, which may not be the case. Hence, future studies should control for smartphone-based hedonic Internet use. Finally, estimates of the percent of the time for hedonic use may only be approximate due to memory bias.

## 5 Conclusions

This study demonstrated that hedonic smartphone use and perceived stress are directly associated with SA. Additionally, satisfaction with life negatively affected SA through its relationship with perceived stress. Finally, the female gender was a direct positive predictor of SA. There was a weak correlation between age, SA, and hedonic smartphone use in this study, but age did not emerge as a predictor of SA. The practical implication of these results is that treatments aimed at the symptoms of SA should assess life stress and the purpose of smartphone use while considering the gender and life satisfaction of the affected person. We also suggest that researchers allow their participants to appraise *what they consider hedonic or utilitarian smartphone use* because measuring application use frequency or duration could be erroneous since numerous applications fulfill both purposes.

## Declaration of Competing Interest

The authors declare that they have no known competing financial interests or personal relationships that could have appeared to influence the work reported in this paper. The study received no external financial support; its operational cost was covered by ELTE Eötvös Loránd University, Budapest, Hungary.
